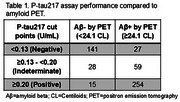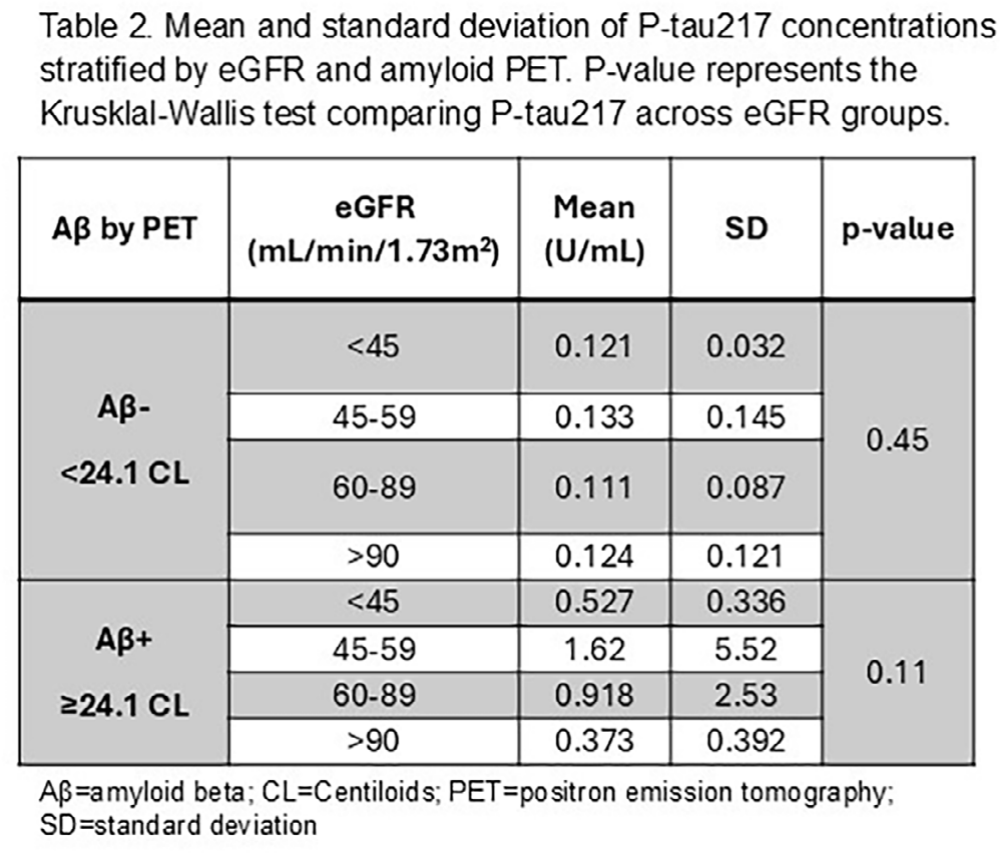# Clinical validation of a plasma *p* ‐tau217 immunoassay and evaluation of the impact of kidney function

**DOI:** 10.1002/alz70861_108683

**Published:** 2025-12-23

**Authors:** Heather A Nelson, Sonia L La’ulu, J Alan Erickson, Sierra Cunningham, Kelly Doyle

**Affiliations:** ^1^ University of Utah Health, Salt Lake City, UT USA; ^2^ ARUP Institute for Clinical and Experimental Pathology, Salt Lake City, UT USA; ^3^ ARUP Institute for Research and Innovation in Diagnostic and Precision Medicine, Salt Lake City, UT USA

## Abstract

**Background:**

Blood‐based biomarkers have emerged as an accessible and lower cost alternative tool to support the diagnosis of Alzheimer disease. This study evaluated the clinical performance of a plasma *p* ‐tau217 immunoassay for identifying AD pathology as detected by amyloid positron emission tomography (PET). The relationship of *p* ‐tau 217 concentration with estimated glomerular filtration rate (eGFR) in individuals with and without reduced kidney function was also investigated.

**Methods:**

Plasma samples from individuals in the Eli Lilly and Company TRAILBLAZER‐ALZ 2 (I5T‐MC‐AACI) trial were analyzed for *p* ‐tau217 using a chemiluminescent immunoassay on the Quanterix SP‐X platform. The performance of plasma *p* ‐tau217 was assessed by comparing *p* ‐tau217 concentration to amyloid PET results, with amyloid PET positivity (Aβ+) defined as 24.1 Centiloids. This data was used to derive two cut points, yielding a sensitivity and specificity ≥90%, with <20% of results falling in the indeterminate zone between cut points.

**Results:**

Samples from 524 individuals (median age 74 years, 52% female, 64.9% Aβ+) were evaluated. Additionally, 78 (14.9%) participants had an eGFR <60 mL/min/1.73m^2^. The area under the receiver operating characteristic curve of plasma *p* ‐tau217 was 0.941. The two cut points established resulted in a sensitivity of 90%, specificity of 90%, negative predictive value of 84%, positive predictive value of 94%, and a percent indeterminate of 17%, with a false positive rate of 2.9% and false negative rate of 5.2% (Table 1). When *p* ‐tau217 concentrations were stratified by Aβ+ and eGFR status, there was no significant change in *p* ‐tau217 in individuals with reduced eGFR compared to those with normal kidney function in amyloid negative (*p* ‐value=0.45, Kruskal‐Wallis‐test) or amyloid positive (*p* ‐value=0.11, Kruskal‐Wallis‐test) individuals (Table 2).

**Conclusion:**

This plasma *p* ‐tau217 assay has high concordance with amyloid PET and may aid in the diagnostic workup of AD. Furthermore, quantification of *p* ‐tau217 using this assay was not impacted by reduced eGFR status, suggesting it can provide reliable results in individuals with renal impairment.